# Droxidopa in Critical Care: A Systematic Review of an Emerging Off‐Label Practice

**DOI:** 10.1155/ccrp/4830160

**Published:** 2025-12-15

**Authors:** Carlos Valladares, Franklyn Vega Batista, Marc Faltas, Mareya Menteyas, Katherine Chiapaikeo-Poco

**Affiliations:** ^1^ Department of Pulmonary and Critical Care, University of Miami, Miami, 33136, Florida, USA, miami.edu; ^2^ Department of Critical Care Medicine, Rutgers Health-RWJ Barnabas Health, Community Medical Center, Toms River, 08755, New Jersey, USA; ^3^ Department of Medicine, Rowan University School of Osteopathic Medicine, Stratford, 08043, New Jersey, USA, rowan.edu

## Abstract

**Background:**

Prolonged use of intravenous (IV) vasopressors in critically ill patients is associated with significant complications. Droxidopa, a norepinephrine precursor approved for neurogenic orthostatic hypotension, has gained interest as an off‐label agent for facilitating vasopressor weaning in ICU settings. This systematic review aimed to evaluate the efficacy and safety of droxidopa for vasopressor weaning in ICU patients.

**Methods:**

A systematic review was conducted following PRISMA guidelines. Databases searched included PubMed, Embase, Cochrane, and Web of Science through April 2025. Eligible studies reporting primary clinical data on droxidopa use in ICU patients were included. Outcomes included time to IV vasopressor discontinuation, duration of droxidopa use, ICU length of stay (LOS), and ICU mortality, and results were narratively synthesized. The risk of bias was assessed using the ROBINS‐I and the JBI checklists.

**Results:**

Seven studies involving 161 ICU patients were included. Five studies reported time to vasopressor discontinuation, ranging from 29 to 120 h. The duration of droxidopa use ranged from 87 to 192 h. Two studies reported ICU LOS, ranging from 18 to 44 days. ICU mortality was inconsistently reported. These findings are primarily drawn from small, retrospective studies and should be interpreted cautiously.

**Discussion:**

Findings suggest that droxidopa may effectively facilitate vasopressor weaning in critically ill patients. However, variations in dosing, patient selection, and outcome reporting limit generalizability. Evidence is drawn primarily from small, retrospective studies, some available only as abstracts.

**Conclusion:**

Available evidence on droxidopa for vasopressor weaning in ICU patients remains limited and heterogeneous, with very low certainty. Further research is warranted. No funding was received for this review, and the review was not prospectively registered.

## 1. Background

Prolonged dependence on intravenous (IV) vasopressors in critically ill patients is more than a hemodynamic challenge. It is associated with delayed recovery and significant clinical risk. This includes ischemic complications, arrhythmias, and catheter‐related infections. These complications can lead to delayed ICU discharge, longer hospital stays, and higher medical expenses [1–4]. As a result, optimizing strategies to transition patients off IV vasopressors is a key goal in intensive care medicine.

Midodrine, an oral alpha‐1 adrenergic agonist, has been widely used off‐label to decrease vasopressor needs. However, its clinical benefit in critically ill populations remains inconsistent, with limited hemodynamic benefit and adverse reactions such as reflex bradycardia reported in several studies [5–8]. This inconsistency has prompted interest in alternative oral agents with more predictable pharmacodynamics.

Droxidopa (L‐threo‐3,4‐dihydroxyphenylserine [L‐DOPS]), a synthetic precursor to norepinephrine, is FDA‐approved for treating neurogenic orthostatic hypotension [9]. It is orally bioavailable and acts by increasing peripheral vascular resistance, making it a physiologically plausible agent for patients with vasodilatory shock [10]. After oral administration, it is absorbed in the small intestine via neutral amino acid transporters and converted to norepinephrine by the enzyme aromatic L‐amino acid decarboxylase (AADC), primarily within sympathetic nerve terminals, vascular smooth muscle, and peripheral tissues. Peak plasma concentrations are typically reached within 3 hours, with a half‐life ranging from two to 3 hours. Pharmacodynamically, droxidopa raises blood pressure through increased systemic vascular resistance, enhancing venous return and cardiac output without directly stimulating adrenergic receptors. Its adverse effects include headache, hypertension, and, less frequently, arrhythmia or ischemia due to catecholamine excess [9–13].

Understanding droxidopa’s pharmacologic profile provides context for its comparison with existing oral vasopressor agents. Compared to midodrine, a selective alpha‐1 agonist, droxidopa offers a unique mechanism by augmenting endogenous norepinephrine synthesis rather than directly activating adrenergic receptors. This distinction may allow for more physiologic vasopressor support and a lower incidence of reflex bradycardia, a known limitation of midodrine therapy [10–13].

Recently, droxidopa has gained popularity among intensivists as an off‐label option to aid in the discontinuation of IV vasopressors in ICU patients. Clinicians have increasingly explored droxidopa as a potential enteral agent for critically ill patients requiring vasopressor support. Despite the absence of formal approval for these indications, it is being utilized across a range of conditions, including septic shock, postcardiac surgery vasoplegia, and spinal cord injury with autonomic dysfunctions [10–13].

Nevertheless, the supporting evidence for droxidopa use remains largely limited to small retrospective studies, case series, and case reports, with considerable heterogeneity in patient selection, dosing strategies, and outcome definitions. These observational studies show that droxidopa treatment can lead to decreased norepinephrine equivalents while shortening vasopressor discontinuation times, thus demonstrating its potential as a critical care medication [11, 14]. To date, no systematic synthesis of the available evidence has assessed the impact of droxidopa on outcomes such as ICU vasopressor discontinuation, hospital length of stay (LOS), or mortality in critically ill patients.

This systematic review aims to address the current evidence gap by evaluating the efficacy and safety of droxidopa in ICU settings. Specifically, we assess its impact on IV vasopressor discontinuation, hospital LOS, duration of droxidopa therapy, and ICU mortality while considering the safety and applicability of its off‐label use in ICU settings.

## 2. Methods

A systematic review was conducted using the Preferred Reporting Items for Systematic Reviews and Meta‐Analyses (PRISMA 2020) guidelines [15]. This review was not prospectively registered in PROSPERO or OSF. The absence of prior registration represents a methodological limitation and raises the possibility of selection or reporting bias. The completed PRISMA 2020 checklist is provided in Supporting information (available here).

### 2.1. Inclusion and Exclusion Criteria

The inclusion criteria included studies that consisted of primary clinical data on the use of droxidopa in the management of ICU patients; retrospective, case series and case‐control studies; and retrospective study abstracts due to the limited data. Outcomes of interest include time to vasopressor discontinuation, droxidopa use duration, ICU LOS, and ICU mortality.

Articles were excluded from analysis if they were case reports that did not contain droxidopa use or ICU patients. Articles were not excluded based on nation of origin. Articles were excluded if they were not in English. Studies were not differentiated based on the dose of droxidopa used. Articles with ongoing studies, no reported data, or animal studies were also excluded. Conference abstracts were retained when sufficient methodological and clinical data were available, given the limited number of peer‐reviewed studies on this topic. A sensitivity narrative review excluding abstracts produced similar qualitative conclusions.

### 2.2. Information Sources and Search Strategy

This systematic review utilized five medical databases to search for articles on the use of droxidopa in ICU units. These databases included PubMed, Embase, Scopus, Web of Science, and the Cochrane Library. Search terms utilized were as follows: (“ICU” OR “Intensive care unit” OR “critical care” OR “critically ill”) AND (“droxidopa” OR “threo‐DOPS” OR “3,4‐threo‐DOPS” OR “3,4‐Dihydroxyphenylserine” OR “norepinephrine precursor” OR “L‐DOPS”). The initial article search was conducted by CV on February 21, 2025. Searches of gray literature in Google Scholar and WorldCat were also performed. The full search string, databases, and search date are detailed above. No language or date limits were applied to ensure comprehensive coverage of the available literature. Duplicate studies were identified and resolved utilizing Rayyan.ai online software [16]. Following the identification of duplicate articles, two reviewers (CV and FV) manually sorted through the retrieved articles to ensure no further duplicates existed; two duplicates were identified and removed.

### 2.3. Study Selection

Once duplicates were identified and sorted through, title and abstract analysis were conducted to determine inclusion. Following the title and abstract analysis, a full‐text appraisal was completed by two trained reviewers (CV and FV). In the event of a debate over the inclusion of a study, a third reviewer was brought in to break ties. Studies determined to be eligible for data analysis (both quantitative and qualitative) were subjected to data extraction.

### 2.4. Data Collection and Analysis

Full‐text appraisal was performed through an initial critical appraisal, followed by data extraction. These data were then examined for relevance, significance, and generalizability. The primary measure the reviewers extracted was the time to vasopressor discontinuation, followed by ICU LOS, duration of droxidopa use, and ICU mortality. They were then subjected to a critique of design.

### 2.5. Certainty of Evidence and Risk of Bias Assessment

Risk of bias was independently assessed by two trained authors (MF and MM) using tools appropriate to each study design. For all retrospective cohort studies, including those available only as conference abstracts with sufficient methodological detail, the ROBINS‐I tool was applied [17]. Data were reported graphically using the ROBVIS stoplight plot and summary chart creation tool [18]. For the included case series, we used the JBI Critical Appraisal Checklist for Case Series, which evaluates methodological quality across 10 criteria, including inclusion criteria, consistent measurement, and outcome reporting [19]. Abstracts without sufficient detail for domain‐level assessment were judged to have a serious risk of bias by narrative review. Disagreements between reviewers were resolved by consensus, and no third‐party adjudication was required. Following quality assessment and data extraction, the findings from the included studies are summarized as follows.

## 3. Results

**Figure 1:**
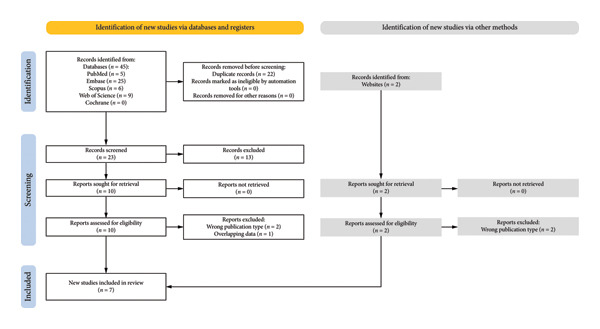
PRISMA 2020 guidelines article selection flowsheet [15].

Querying of 5 databases yielded a total of 45 articles. Detection of duplicates via Rayyan.ai’s automatic function and subsequent removal of 22 articles yielded 23 original articles. Articles were then subjected to abstract and title appraisal. Out of the 23 articles, only 10 remained. A full‐text appraisal was then conducted on the 10 articles. Of the articles subjected to full‐text appraisal, 2 were excluded from the final analysis as they were the wrong publication type [13, 20]. This meant the articles were not RCT, retrospective, case‐control, or case series. One was excluded as it had overlapping data [21]. Gray literature searches on Google Scholar and WorldCat yielded 2 articles that were retrieved for full‐text appraisal but were ultimately removed as they were the wrong publication type [12, 22]. Ultimately, 7 articles satisfied the inclusion criteria (Figure 1). Data were then extracted and analyzed from the included articles involving a total of 161 patients. A summary of the included articles can be seen in Table 1 [11, 14, 23–27].

**Table 1:** Summary of included articles.

Study (Year)	Study design	Sample size (*n*)	ICU type	Baseline IV vasopressor (*s*)	Droxidopa regimen (start ⟶ max)
Lessing et al. [11]	Retrospective single‐center study	45 (27 received droxidopa)	Cardiothoracic ICU	Norepinephrine ± vasopressin	100 mg·TID ⟶ 600 mg·TID
Webb et al. [14]	Multicenter retrospective	30	Medical‐surgical ICU	Norepinephrine ± vasopressin	100 mg·TID ⟶ 2400 mg/day
Noble et al. [23]	Single‐center retrospective	18	Mixed ICU	Norepinephrine	100 mg·BID ⟶ 500 mg·TID
Ying et al. [24]	Multicenter retrospective	50	Medical ICU	Norepinephrine	100–300 mg
Diep et al. [25]	Single‐center retrospective study	33	Mixed ICU	Norepinephrine ± vasopressin	100 mg·TID ⟶ 600 mg·TID
Hong et al. [26]	Case series (2 cases)	2	Neurosurgical ICU	Norepinephrine	Case 1: 100 mg·TID; Case 2: 100 mg·BID ⟶ 600 mg TID
Oommen et al. [27]	Case series (2 cases, 1 ICU included)	1 (ICU case only)	Surgical ICU	Norepinephrine ± vasopressin	100 mg·TID ⟶ 300 mg·TID

### 3.1. Full‐Text Articles Excluded From Analysis

Of the articles subjected to full‐text appraisal, five were excluded from the final analysis [12, 13, 20–22]. While some studies presented challenges during screening, such as being nonpeer review abstracts, they were still retained due to the limited availability of literature on this topic. Given the emerging nature of droxidopa use in critically ill patients, even studies with less conventional formats were considered valuable for inclusion, provided they offered relevant clinical data. The study by Oommen et al. [27] was included because one of the two reported cases involved a critically ill patient in the surgical intensive care unit who required IV norepinephrine and vasopressin before and during droxidopa therapy. Although the other case described outpatient management of orthostatic hypotension without vasopressors, inclusion was justified as the ICU case met the eligibility criteria and provided valuable safety and dosing data relevant to droxidopa use in critically ill patients.

### 3.2. Results of Quality of Evidence Rating and Risk of Bias Assessment

**Figure 2:**
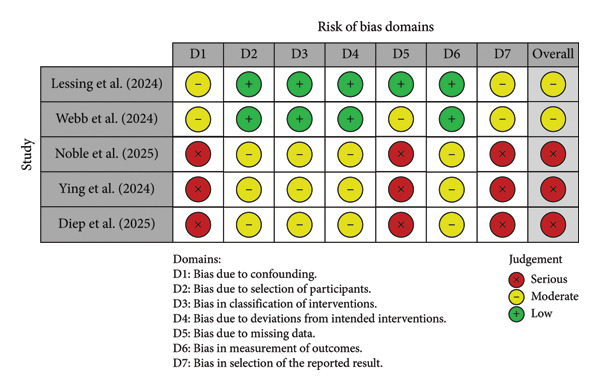
ROBINS‐I domain‐level risk of bias summary for retrospective studies.

**Figure 3:**
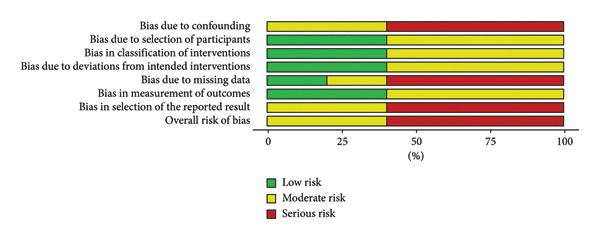
Overall risk of bias summary using the ROBINS‐I tool.

Bias was independently appraised based on their respective study design. Retrospective cohort studies, including those reported as conference abstracts with sufficient methodological detail, were evaluated using the ROBINS‐I tool. Most retrospective studies demonstrated a serious risk of bias, particularly in confounding, missing data, and outcome measurement domains, as summarized in Figures 2 and 3 and detailed in Table 2. Data were reported graphically using the ROBVIZ stoplight plot and summary chart creation tool (Figures 2 and 3) [17].

**Table 2:** Summary of risk of bias assessment using the JBI checklist for included case series.

Study	Inclusion criteria defined	Consistent measurement	Complete follow‐up	Overall appraisal
Oomen et al. [27]	No	Yes	No	Include with caution
Hong et al. [26]	Yes	Yes	Yes	Include

Case series were assessed using the JBI Critical Appraisal Checklist for Case Series and were deemed appropriate for inclusion [19]. Results are summarized in Table 2. Disagreements between the two authors’ decisions were to be settled by a third author, but this was not needed.

### 3.3. Overview of Outcomes

Of the included articles, a total of 161 patients were identified. 30 were reported to be female (18.6%), 48 male (29.8%), and 83 of unknown sex (51.6%). Patient ages ranged from 61 to 75 years old. The average age was 66.2 (median = 61). All of the patients were critically ill and required ICU care. During their hospitalization process, all the patients were started on droxidopa to aid with vasopressor weaning. Other outcomes including ICU LOS, time to vasopressor discontinuation, duration of droxidopa use, and ICU mortality were also explored. A comprehensive table of the studies, and their outcomes, is presented in Supporting Table S1.

### 3.4. Time to Vasopressor Discontinuation

Time to IV vasopressor weaning following droxidopa initiation was reported in five studies. The shortest median weaning time was reported by Ying et al. at 29 h, and Webb et al. and Diep et al. observed median times of 70 and 72 h, respectively [14, 24, 25]. Lessing et al. reported a slightly longer duration of 5 days (∼120 h) [11]. Hong et al. qualitatively reported successful weaning off IV vasopressors, without a precise time frame [26]. Definitions of “vasopressor discontinuation” varied: some defined it as the first 24‐h period without IV vasopressors [14, 24, 25], while others did not specify the criteria [11, 23, 26, 27]. This variability limits comparability across studies.

### 3.5. Duration of Droxidopa Use

The duration of droxidopa therapy was variably reported across studies. Webb et al. reported a median duration of 87 h, while Lessing et al. reported 5 days [11, 14]. Diep et al. described a median duration of 8 days, and Oomen et al. reported 48 h of treatment [25, 27]. In Noble et al. and Hong et al., droxidopa use was mentioned, but no specific duration was reported [23, 26]. While Ying et al. did not report this outcome [24].

### 3.6. ICU LOS

ICU LOS was reported in two of the included studies. Webb et al. observed the longest median ICU LOS at 44 days in a mixed ICU population, while Lessing et al. reported a median ICU LOS of 18 days among cardiothoracic ICU patients [11, 14]. The remaining studies, including Noble et al. and Oomen et al., did not report ICU LOS [23–27].

### 3.7. ICU Mortality

ICU mortality was not frequently reported. Webb et al. observed a mortality rate of 47% among their ICU cohort [14]. Lessing et al. reported no significant difference in ICU mortality between droxidopa‐treated patients and controls [11]. The remaining studies (Noble et al. and Oomen et al.) did not report ICU mortality outcomes [23–27].

## 4. Discussion

This systematic review included seven studies comprising 161 critically ill ICU patients who received droxidopa as an adjunct to facilitate IV vasopressor weaning. Most patients were between 51 and 75 years old, with limited reporting of sex and comorbidities. Across five studies reporting this outcome, median time to vasopressor discontinuation ranged from 29 to 120 h. Droxidopa use duration was reported in four studies and ranged from 87 to 192 h. ICU LOS was available in only two studies (18–44 days), and ICU mortality was inconsistently documented, with one study reporting a 47% rate.

Despite variation in study design and reporting, droxidopa was generally associated with successful vasopressor discontinuation within 72 h. However, differences in patient selection, timing of droxidopa initiation, dosing strategies, and outcome definitions limit comparability. While the overall data suggest that droxidopa may support vasopressor weaning in selected ICU populations, the evidence is limited by small sample sizes, heterogeneity in the study designs, and incomplete outcome reporting.

### 4.1. Time to Vasopressor Discontinuation

This outcome was reported in five studies. Median times ranged from 29 h in Ying et al. to 120 h in Lessing et al., with most studies observing discontinuation within 72 h of droxidopa initiation [11, 24]. These findings suggest a potential role for droxidopa in facilitating hemodynamic stabilization. However, the timing of initiation relative to vasopressor onset varied and was not always clearly reported, which complicates the interpretation of efficacy [14, 23, 25].

### 4.2. Duration of Droxidopa Use

The reported length of droxidopa was inconsistently reported. Webb et al. described a median duration of 87 h, Diep et al. reported 8 days (192 h), and Lessing et al. noted a 5‐day median [11, 14, 25]. The studies by Hong et al. and Noble et al. referenced the use of droxidopa but did not provide specific treatment durations [23, 26]. Ying et al. did not report this outcome [24]. These variations reflect differing institutional protocols and thresholds for discontinuation, which could influence both perceived effectiveness and clinical decision‐making.

### 4.3. ICU LOS

Only two studies reported this outcome, limiting comprehensive analysis. Webb et al. documented a prolonged median ICU LOS of 44 days in a mixed ICU population, whereas Lessing et al. reported a shorter median ICU LOS of 18 days among cardiothoracic surgery patients [11, 14]. This difference likely reflects variability in baseline patient acuity and ICU type. The remaining studies either did not report ICU LOS or lacked sufficient follow‐up.

### 4.4. ICU Mortality

Mortality data were reported in only two studies, limiting interpretation. Webb et al. noted an ICU mortality rate of 47% [14]. Lessing et al. stated that there was no significant difference in mortality between droxidopa‐treated patients and controls, though exact rates were not disclosed [11]. The remaining studies either did not report mortality outcomes or had incomplete follow‐up, which limits conclusions about survival benefit.

### 4.5. Route of Administration

Droxidopa was administered either orally or via enteral feeding tubes in intubated ICU patients. Available studies suggest that enteral administration achieves comparable hemodynamic responses to oral dosing, supporting its feasibility in mechanically ventilated patients unable to take medications by mouth [21, 22, 26]. Absorption through the gastrointestinal tract remains adequate as long as bowel perfusion and motility are preserved. However, no study to date has compared pharmacokinetic profiles or norepinephrine level changes between oral and gastric administration. This knowledge gap underscores the need for standardized administration protocols, as impaired absorption in critically ill patients could alter therapeutic efficacy.

### 4.6. Dosing Variability and Timing

Dosing regimens varied widely, reflecting the lack of standardized ICU protocols. Most studies initiated droxidopa at 100 mg twice daily and titrated to 400–600 mg three times daily, with maximum doses reaching 2400 mg/day [11, 14, 23]. In several cases, droxidopa was introduced after failed midodrine therapy or in combination with it [24, 25]. In the cardiothoracic ICU cohort by Lessing et al., droxidopa was combined with atomoxetine, a norepinephrine reuptake inhibitor, to enhance endogenous catecholamine levels [11]. Timing of initiation also differed: some centers administered droxidopa once norepinephrine requirements were stable but persistent, whereas others started it earlier to expedite weaning. The absence of consistent initiation thresholds and tapering schedules makes it difficult to determine the optimal dosing strategy or timing for maximal benefit.

### 4.7. Safety Profile

Adverse events were sparsely reported. Only two studies mentioned complications, including transient hypertension, bradycardia, or tachyarrhythmia [11, 14]. No ischemic events were observed, but incomplete reporting limits interpretation. Given droxidopa’s catecholaminergic mechanism, cardiovascular monitoring is essential in future studies to establish a clearer safety profile.

Taken together, these findings highlight significant variability in study design and patient characteristics, underscoring the need for standardized protocols and better‐quality evidence.

### 4.8. Limitations

This systematic review is not without limitations. One major limitation is the heterogeneity of included patient populations and comorbidities, which obscures the interpretation of droxidopa’s effectiveness. Patients often differed widely in baseline disease severity, including cardiac dysfunction, renal failure, or sepsis, all of which can prolong ICU stay and affect vasopressor requirements independent of droxidopa use.

Additional limitations include the small sample size and heterogeneity across the included studies, which reduce the overall strength of the evidence. Most of the included studies were retrospective cohorts or case series, all of which are considered low or very low quality. Only a few studies incorporated comparator groups or standardized outcome measurements, and most lacked details on exact droxidopa dosing schedules, timing of initiation, or vasopressor weaning protocols. Outcome definitions, including time to vasopressor discontinuation and ICU mortality, were inconsistently defined across studies, leading to uncertainty in denominators and limiting pooled interpretation.

Furthermore, several of the included studies were available only as conference abstracts, so publication bias and reporting bias cannot be ruled out. The studies spanned different years and ICU practices, introducing temporal variability in patient management. The lack of standardized definitions for vasopressor dependence or weaning success complicates attempts to synthesize the data meaningfully.

Finally, the ROBINS‐I risk of bias assessment showed that several studies did not provide follow‐up duration or postdischarge outcomes. This was particularly noticeable in single‐arm observational studies and case series, many of which did not present consistent mortality or survival metrics. Together, these methodological gaps underscore the very low certainty of current evidence and highlight the urgent need for prospective, well‐controlled studies to evaluate droxidopa’s true efficacy and safety profile in the ICU setting.

## 5. Conclusion

The current evidence regarding droxidopa use for vasopressor weaning in ICU patients remains limited and is of very low certainty. Observational data suggest a possible benefit in facilitating vasopressor discontinuation, but these findings are hypothesis‐generating and not definitive. Prospective, controlled studies are urgently needed to confirm efficacy, safety, and optimal dosing strategies before routine use can be recommended.

### 5.1. Future Considerations

Future studies should overcome the current limitations in the literature by using larger sample sizes, well‐defined treatment guidelines, and comparison groups of standard care. To accurately determine the real effect of droxidopa in reducing ICU LOS and improving clinical outcomes, further research is needed using randomized controlled trials. These controlled trials should focus on the optimal timing of administration of droxidopa, dosing protocols, and patient selection criteria, including an analysis of comorbidities and baseline vasopressor needs. Further research should also explore whether specific patient populations, such as those with minimal cardiovascular and renal comorbidities, may benefit more from droxidopa treatment.

**Nomenclature**
ICU: Intensive care unit
IV: Intravenous
LOS: Length of stay

## Conflicts of Interest

The authors declare no conflicts of interest.

## Author Contributions

Carlos Valladares: original draft, literature screening, data extraction, interpretation, manuscript editing, and revision. Franklyn Vega Batista: data extraction, literature screening, and manuscript editing. Marc Faltas: formal analysis, figure preparation, and manuscript revision. Mareya Menteyas: risk of bias assessment, quality control, methodology, and validation. Katherine Chiapaikeo‐Poco: manuscript editing and revision.

## Funding

No funding was received for this study.

## Supporting Information

Supporting Information. Table S1 includes a summary of all the studies and outcomes.

## Supporting information


**Supporting Information** Additional supporting information can be found online in the Supporting Information section.

## Data Availability

The data that support the findings of this study are available from the corresponding author upon reasonable request.
