# Supplementary material for: Droxidopa in Critical Care: A Systematic Review of an Emerging Off‐Label Practice
Source: Crit Care Res Pract. 2025 Dec 15;2025:4830160. doi: 10.1155/ccrp/4830160 (PMC12714080; doi:10.1155/ccrp/4830160)
Supplement: Supplementary file 1 — Supporting Information Additional supporting information can be found online in the Supporting Information section. [file CCRP-2025-4830160-s001.pdf]

Table S1. Included studies and outcome overview

Study (Year)	Study Design	Sample Size (n)	ICU Type	Baseline IV Vasopressor(s)	Droxidopa Regimen (Start → Max)	Timing of Initiation	Co-therapies (Midodrine / Others)	Weaning Time	ICU LOS	Mortality	Adverse Events Reported
**Lessing et al., 2024 [11]**	Retrospective single-center study	45 (27 received droxidopa)	Cardiothoracic ICU	Norepinephrine ± vasopressin	100 mg TID → 600 mg TID	After midodrine or atomoxetine failure	Midodrine ± atomoxetine	≈ 8 days (median)	18 days	Not reported	None reported
**Webb et al., 2025 [14]**	Multicenter retrospective	30	Medical-surgical ICU	Norepinephrine ± vasopressin	100 mg TID → 2400 mg/day	Median 16 days after vasopressor start	Midodrine ± fludrocortisone ± pseudoephedrine	70 h (median 23–192 h)	44 days	47 % (in-hospital)	Tachycardia (13 %); no ischemia
**Noble et al., 2025 [24]**	Single-center retrospective	18	Mixed ICU	Norepinephrine	100 mg BID → 500 mg TID	During persistent hypotension	Midodrine (100 %)	No significant change reported	Not reported	Not reported	None reported
**Ying et al., 2024 [23]**	Multicenter retrospective	50	Medical ICU	Norepinephrine	100–300 mg	During recovery phase	Midodrine (32 %)	29 h	Not reported	9 deaths (18 %)	Tachycardia (2); delirium (1)
**Diep et al., 2025 [25]**	Single-center retrospective study	33	Mixed ICU	Norepinephrine ± vasopressin	100 mg TID → 600 mg TID	≈ 25 days post-ICU admission	Midodrine (91 %); steroids (55 %)	40 % off pressors within 72 h	Not reported	Not reported	None reported
**Hong et al., 2022 [26]**	Case series (2 cases)	2	Neurosurgical ICU	Norepinephrine	Case 1: 100 mg TID; Case 2: 100 mg BID → 600 mg TID	During weaning phase after midodrine bradycardia	Midodrine ± pseudoephedrine	[illegible]ase 1); 9 days (case 2)	Not reported	Not reported	Transient tachycardia (case 2)
**Oommen et al., 2019 [27]**	Case series (2 cases, 1 ICU included)	1 (ICU case only)	Surgical ICU	Norepinephrine ± vasopressin	100 mg TID → 300 mg TID	After midodrine failure (day 35 ICU)	Midodrine ± fludrocortisone	Not reported	Not reported	Not reported	Tachycardia (HR > 120 bpm)

1. NE = norepinephrine; LOS = length of stay; h = hours
2. Numbers in parentheses indicate the number of patients (for adverse events) or percentage of the cohort (for co-therapies) as reported by each study.
3. Only the ICU case from Oommen et al. met inclusion criteria; the outpatient orthostatic case was excluded.
4. “Not reported” indicates data not provided in the source study.
